# Ethnicity modifies the relationship between added sugars and fructose exposure in the first 1000 days and offspring body composition at 24 months

**DOI:** 10.1016/j.nutres.2025.04.009

**Published:** 2025-04-19

**Authors:** Sara A. Fortin-Miller, Byron J. Gajewski, Susan E. Carlson, John A. Colombo, Danielle N. Christifano, Debra K. Sullivan, Holly R. Hull

**Affiliations:** a Department of Dietetics and Nutrition, University of Kansas Medical Center, Kansas City, KS, USA; b Department of Biostatistics and Data Science, University of Kansas Medical Center, Kansas City, KS, USA; c Department of Psychology and Schiefelbusch Institute for Life Span Studies, University of Kansas, Lawrence, KS, USA; d Department of Obstetrics and Gynecology, University of Kansas Medical Center, Kansas City, KS, USA

**Keywords:** Pregnancy, Infancy, Toddlerhood, 1000 days, Added sugars, Fructose, Hispanic, Adiposity

## Abstract

Added sugars (AS) and fructose intake are linked to obesity. Hispanic populations experience high AS intake and obesity rates. It is underexplored if early sugars exposure influences offspring growth, especially across ethnic groups. This secondary analysis examined if AS and fructose intake during pregnancy, infancy, and toddlerhood influenced offspring adiposity at 24 months, and if ethnicity modified outcomes. We hypothesized that higher sugars exposure would predict increased adiposity. Mother-child pairs from a prenatal docosahexaenoic acid supplementation RCT and the offspring follow-up study were included. Dietary intake was assessed at 12 to 20 weeks gestation, and offspring intake at 2 weeks, 6 months, 12 months, and 24 months. Intake was averaged across infancy (Year 1: 2 weeks/6 months) and toddlerhood (Year 2: 12 months/24 months). Anthropometric (*n* = 130) and dual-energy X-ray absorptiometry (*n* = 42) data were collected at 24 months. Multiple hierarchical regression examined associations, with interaction terms testing ethnic differences. Higher AS intake in Year 1 predicted higher weight, fat-free mass, and total adiposity. Compared to non-Hispanic offspring, the association of Year 1 fructose intake with weight and Year 2 AS intake with fat-free mass and central fat mass, were weaker in Hispanic offspring. No other significant associations were observed. The relationships between early AS and fructose intake with body composition are dynamic over time and vary by ethnicity. Our findings highlight the potential risks of early sugars exposure on obesity and metabolic health, underscoring the need for further research to inform early-life dietary interventions and public health policies.

## 1. Introduction

The first 1000 days, spanning from conception to 24 months old, is a critical period during which the foundations of health across the lifespan are established [1]. Nutritional needs during this period are maximized to support optimal growth and development [2], which leaves little room for foods that provide empty calories. Empty calories are calories derived from foods that contain minimal nutrient value, including foods and beverages high in added sugars (AS).

Six monosaccharides and disaccharides represent AS—glucose, fructose, galactose, sucrose, lactose, and maltose. The definition of AS is “all sugars, syrups, or caloric sweeteners added to food during processing, production, and/or cooking” [3]. Habitual intake of AS is associated with an increased risk of obesity and poor health outcomes [4]. Fructose is particularly considered a driver of obesity due to its (1) unregulated metabolism compared to glucose and (2) overabundance in the food supply mainly from sugar-sweetened beverages (SSB) or common sweeteners including corn syrup solids.

The Dietary Guidelines for Americans (DGA) recommend limiting AS intake to less than 10% of daily calories for pregnant and nonpregnant individuals ≥24 months old, and that infants and toddlers < 24 months consume *no* AS [5]. Intake of AS during the first 1000 days is high accounting for ~14.8% of total daily energy intake in pregnant women [6], with 70% of pregnant women [5] and 84.4% of infants and toddlers consuming AS daily [7]. AS intake during pregnancy is positively associated with offspring weight and adiposity at 6 months [8], and weight status at 2 to 4 years [9]. In older children (2–11 years) [4, 10] and adolescents (12–18 years) [4], cross-sectional studies also show a positive association between AS intake and weight status. Limited research has evaluated early fructose intake as a dietary component on offspring growth, though studies link SSB intake during pregnancy to higher offspring adiposity at 6 to 8 years [11, 12], and SSB intake in early childhood (3–11 years) to increased adiposity [10, 13–15]. However, few studies have assessed the relationship between AS and fructose as dietary components during the first 1000 days and offspring adiposity, or if relationships vary by ethnicity.

Compared to other ethnic groups, Hispanic infants often experience different growth trajectories characterized by low birth weight followed by rapid weight gain [16]—a known risk factor for later obesity. Indeed, Hispanic children exhibit higher rates of obesity [16–18] and central obesity [19]. The mechanisms underlying these early-life differences remain unclear. AS intake during infancy and toddlerhood is linked to rapid weight gain [20], but this relationship has not been studied in a Hispanic cohort. AS intake is prevalent among Hispanic pregnant women, children, and adolescents [6, 7, 21, 22]. Therefore, it is important to assess whether ethnicity influences the relationship between early AS and fructose intake and child adiposity.

Our study aimed to investigate the relationship between AS and fructose intake during pregnancy, infancy, and toddlerhood on offspring adiposity at 24 months, and how results vary by ethnicity. We hypothesized that higher AS and fructose intake would predict higher adiposity, with Hispanic offspring showing a stronger association. To test our hypothesis, we assessed dietary data from pregnancy through 24 months and measured offspring body composition at 24 months in a cohort of non-Hispanic and Hispanic mother-child pairs. Nutrition in the first 1000 days plays a foundational role in growth and development. Understanding the impact(s) of early nutritional exposures can improve growth and developmental outcomes and help inform public health policies for better health across generations.

## 2. Methods and materials

This secondary analysis assessed mother-child pairs from two NIH-funded clinical trials—the assessment of docosahexaenoic acid (DHA) on Reducing Early Preterm Birth (ADORE; R01HD83292) and the offspring follow-up study, the Growth and Adiposity in Newborns Study (GAINS; R01DK118220). The ADORE trial has been described in detail elsewhere [23] and the primary results have been published [24]. Briefly, ADORE was a multisite, double-blinded, controlled, Phase III trial in which pregnant women were randomized to either receive 1000 mg/d (intervention) or 200 mg/d (control) of DHA capsules. Women who were 18 years or older, between 12 and 20 weeks gestation, available by phone, English- or Spanish-speaking, and agreed to consume study capsules were eligible to enroll. Women who were pregnant with multiple fetuses, unwilling to discontinue the use of another prenatal DHA supplement, or had an allergy to any component of the DHA product were excluded. Offspring born to women from the ADORE trial were invited to enroll in GAINS to determine how prenatal DHA dose interacted with gestational weight gain (GWG) during pregnancy to influence offspring body composition. The GAINS protocol [25] and the primary results have been published [26]. Of the 489 women who enrolled in ADORE at the Kansas City site, a total of 254 participants enrolled in GAINS. The University of Kansas Medical Center (KUMC) Institutional Review Board reviewed and approved ADORE (STUDY00003455) and GAINS (STUDY00140895). Both trials were registered at clinicaltrials.gov as NCT02626299 (ADORE) and NCT03310983 (GAINS).

### 2.1. Maternal and offspring characteristics

Maternal characteristics including age, race, ethnicity, education, income, and anthropometrics were collected at the ADORE enrollment. Women were asked to self-identify their ethnicity—Hispanic or non-Hispanic. Offspring demographic information was collected at GAINS enrollment. Parents were asked to identify the ethnicity of the child—Hispanic or non-Hispanic. Offspring dietary characteristics including feeding method and feeding mode were collected at each time point—2 weeks, 6 months, 12 months, and 24 months. Anthropometrics and body composition were collected at the 24-month time point.

### 2.2. Maternal and offspring dietary assessment

Maternal dietary intake was measured at ADORE enrollment (12–20 weeks gestation). The National Cancer Institute (NCI) Diet History Questionnaire-II (DHQ-II) food frequency questionnaire (FFQ) was completed by English-speaking non-Hispanic women. Data collected from the DHQ-II was analyzed using Diet*Calc Analysis Software to generate dietary intake of nutrients and food groups. The DHQ-II is not validated for use in the Hispanic population and does not appropriately capture the dietary intake of individuals who do not conform to a traditional US diet. Therefore, Spanish-speaking and English-speaking Hispanic women completed three 24-hour dietary recalls, two during the weekdays and one on the weekend. Trained research staff fluent in Spanish collected the recall information using the multiple-pass method. For the offspring, one multiple-pass 24-hour recall was collected by a registered dietitian at the 2-week, 6-month, 12-month, and 24-month time points. Maternal and child recalls were entered into the Nutrition Data System for Research (NDSR; version 2017, Minneapolis, MN) for macronutrient and micronutrient analysis, including AS and fructose.

The NDSR nutrient output was corrected to quantify the amounts of AS in infant formulas. To meet the nutritional needs of infants, the US Food and Drug Administration (FDA) requires that infant formulas contain certain levels of protein, fat, and certain micronutrients for each 100 calories of formula [27]. However, there are no specifications for the source or amount of carbohydrates. Milk-based infant formulas use lactose to mimic breast milk as closely as possible and to meet the FDA-stipulated amount of calories after protein and fat requirements have been met. If the calorie threshold is still not met, manufacturers may add additional lactose to meet energy specifications. When using the NDSR software to estimate nutrient intake, this additional lactose is counted as AS. Per the definition of AS, lactose when present in milk or dairy products is not considered an AS [28]. Therefore, a data file containing an adjustment factor for each formula used by the GAINS participants was obtained from NDSR. Lactose was subtracted from the AS values and adjusted total AS values were calculated. For lactose-reduced or lactose-free infant formulas, carbohydrates sources are often glucose-derived polymers (e.g., corn syrup, corn syrup solids, brown rice syrup, glucose syrup solids, maltodextrin, sucrose) which, per the definition of AS, were considered sources of AS by NDSR and within our analysis.

### 2.3. Offspring anthropometrics and body composition

Anthropometrics and body composition of the GAINS offspring were assessed at 24 months by trained research staff. Participants were dressed down to a clean diaper and weighed on a standing scale; the infant scale was used if a standing weight could not be obtained. Length was measured using a wall-mounted stadiometer; the length board was used if a standing height could not be obtained. Weight-for-length (WFL) *z*-scores were calculated. A Gulick measuring tape was used to measure waist circumference (WC). A Lange skinfold (SKF) caliper was used to measure SKF thickness at the biceps, thigh, triceps, suprailiac, subscapular, and flank. Peripheral (biceps, thigh, triceps) and central (suprailiac, subscapular, flank) SKF were summed and averaged to assess fat distribution. Dual-energy X-ray absorptiometry (DXA; Prodigy, Madison, WI, encore software version 13.60) was used to measure total body fat and regional adipose tissue distribution. Using specific anatomic landmarks [25], regions including the arms, legs, and trunk were demarcated. Peripheral fat mass (FM) was represented by the sum of the adipose tissue measured in the arms and legs, while the central FM was represented by the adipose tissue measured in the trunk region. Demarcation, along with the usability of each region and the overall scan, were evaluated by a single trained staff member to maintain data integrity. Scans were evaluated for movement to determine the usability of the regions and the overall scan. Scans were then exported, and calculations were completed for percentage body fat (%fat), FM, fat-free mass (FFM), trunk or central FM, and peripheral FM. For this analysis, total body-less head scans were used to calculate all body composition variables.

### 2.4. Statistical analyses

**Fig. 1 –:**
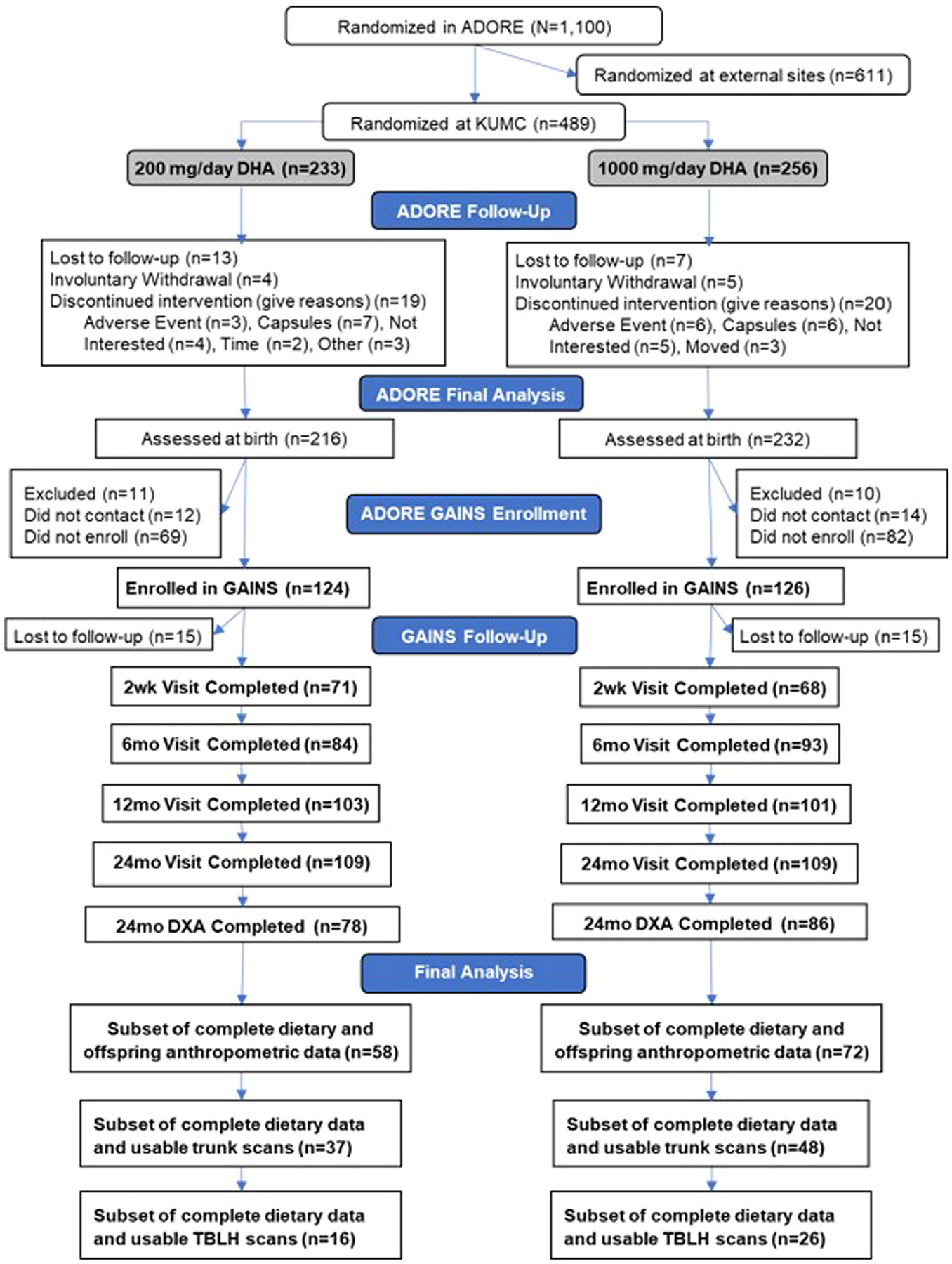
ADORE GAINS consort diagram. A total of 489 mother-child pairs from the ADORE trial enrolled at the KUMC site. Of those pairs, 250 enrolled in GAINS during which data on the offspring were collected at 2 weeks, 6 months, 12 months, and 24 months. For this analysis, 130 mother-child pairs had complete dietary and anthropometric data, 85 pairs had complete dietary and trunk scans, and 42 pairs had complete dietary and TBLH scans. ADORE, Assessment of DHA on Reducing Early Preterm Birth Trial; DHA, docosahexaenoic acid; GAINS, Growth and Adiposity in Newborns Study; KUMC, University of Kansas Medical Center; TBLH, total body-less head.

Sample characteristics were summarized as mean ± SD for continuous variables and frequency (%) for categorical variables. Group differences in continuous variables were analyzed using t-tests, with effect sizes quantified by Cohen’s *d* (small = 0.2, moderate = 0.5, large = 0.8). Chi-squared tests assessed categorical variables, with effect sizes measured by Cramer’s *V*, using the same benchmarks. Fig. 1 shows the flow of participants through ADORE and GAINS by treatment assignment. There were 130 mother-child dyads who had complete dietary and anthropometric data. For our subset analysis, 85 mother-child dyads had complete dietary data and usable trunk scans and 42 had complete dietary data and usable total body-less head scans.

Collection of at least two dietary recalls is considered sufficient for estimating usual energy and nutrient intake [29]. We averaged the offspring recalls collected at 2 weeks and 6 months to represent “Year 1” intake, and the recalls collected at 12 and 24 months represented “Year 2” intake. The decision to average the time points was guided by principal component analysis, using varimax rotation, which confirmed cohesion for these two distinct phases of intake for both AS and fructose. To maintain consistency with the DGA recommendations and optimize translatability, relative intake for both AS and fructose were calculated by multiplying the gram amount of AS and fructose by 4 and then dividing by the mean total energy intake.

Multiple hierarchical linear regression models were completed. The main aim of our project was to assess the relationship between intake of AS and fructose during pregnancy, infancy (Year 1), and toddlerhood (Year 2) and offspring anthropometric measures of adiposity at 24 months (WFL *z*-score, WC, central SKF, and peripheral SKF). In a subset of the sample, we assessed the same relationships using DXA outcomes (%fat, FM, FFM, central FM, peripheral FM). In all models testing the effects of Year 1 and Year 2 AS and fructose intake, an interaction term was included to evaluate if results differed by offspring ethnicity. Since different dietary intake tools were used to assess maternal intake, only the main effect of maternal intake was tested. Given the high collinearity between self-reported maternal and child ethnicity, the ethnicity of the child was used to account for the influence of ethnicity from both parents. Only 4/130 mothers (3%) self-reported a different ethnicity than their child. Maternal covariates included age at enrollment, prepregnancy body mass index (BMI), total GWG, education, smoking history, parity, development of gestational diabetes mellitus or a hypertensive disorder of pregnancy, and DHA treatment group. Offspring covariates included race, sex, gestational age at birth (weeks), and age at the time of measurement. In the fructose models, maternal and offspring fruit servings were included as covariates to account for naturally occurring fructose from fruit intake. To lessen the correlation between the interaction terms and their component variables and to facilitate interpretation of parameter estimates, all continuous predictor variables were centered before regression modeling. A backward selection approach was used to eliminate covariates not statistically contributing to the regression equation (*P* > .10) and to enhance predictive accuracy. Once the model was reduced, nonsignificant interaction terms (*P* > .10) were similarly removed in sequential order. For the DXA analysis, the same approach was used; however, given the smaller sample size, a stricter *P*-value threshold of .05 was applied. Statistical significance was defined as *P* < .05, while .05 ≤ *P* ≤ .10 indicated an approach to statistical significance. All analyses were performed using IBM SPSS, version 29.

## 3. Results

### 3.1. Descriptive characteristics

Maternal and offspring descriptive characteristics are presented in Table 1. Overall, the women in our sample had a mean age of 31.4 years, prepregnancy BMI of 28.2 kg/m^2^, 13.7 kg GWG, and 35.4% self-identified as Hispanic. Further, 62.3% had an overweight/obese prepregnancy BMI and 14.6% developed gestational diabetes mellitus or hypertensive disorder of pregnancy during their pregnancy. For the offspring, average age at the time of the visit was 24.6 months, 50% were male, the majority were white (66.9%) and were born full-term (92.3%; average gestational age at birth 38.7 weeks), and 38.5% were identified as Hispanic. There were maternal differences observed based on ethnicity. Hispanic women gained less weight during pregnancy (−5.43 kg, *P* < .01) and were less likely to gain excessively per GWG guidelines (32.6% Hispanic vs 66.7% non-Hispanic, *P* < .01). Additionally, Hispanic women had less educational attainment, lower household income, and were more likely to have previously given birth (*P* ≤ .01). Since the offspring were born to women who participated in a prenatal supplementation trial, we determined if there were differences in the assignment to the high or low supplementation groups. No differences in group assignment between Hispanic and non-Hispanic offspring were found.

### 3.2. Dietary and body composition characteristics

**Fig. 2 –:**
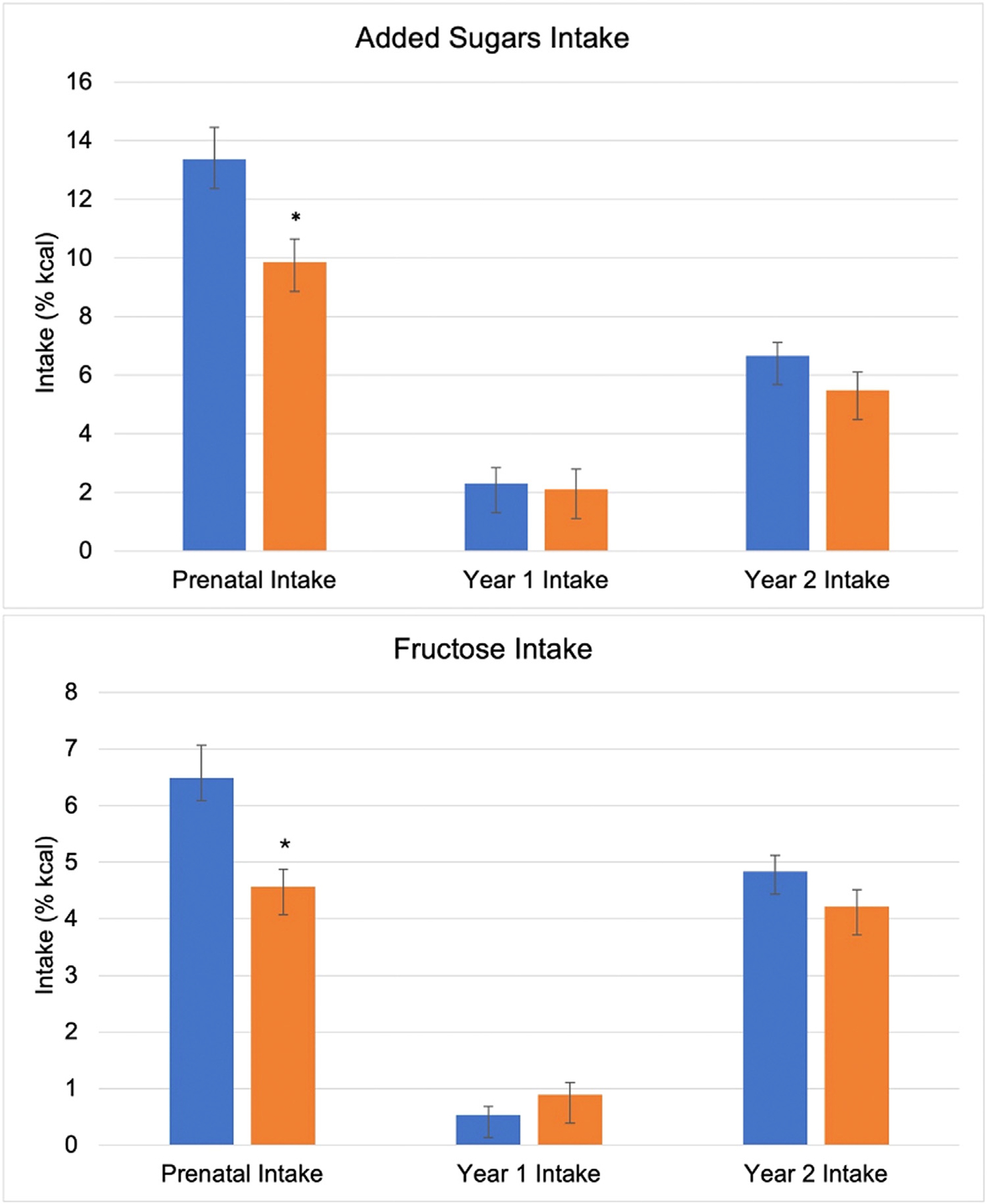
A descriptive comparison of added sugars and fructose intake between ethnic groups. Blue and orange bars illustrate the relative intake of added sugars and fructose in non-Hispanic and Hispanic participants, respectively. *Indicates a significant difference in intake between groups (*P* < .05). Error bars represent mean standard error. Prenatal added sugars intake (%kcal) was lower in Hispanic mothers (vs non-Hispanic). Year 1 and Year 2 added sugars intake did not differ between groups. Prenatal fructose intake (%kcal) was lower in Hispanic mothers compared to non-Hispanic mothers. Offspring fructose intake in Year 1 and Year 2 did not differ between groups.

Maternal and offspring dietary characteristics are presented in Table 2. Descriptive comparison of AS and fructose intake by ethnicity is presented in Fig. 2. In the full cohort, maternal AS intake during pregnancy averaged 12.1% of daily energy. Maternal fructose intake during pregnancy was 5.8% of daily energy. Offspring consumed an average of 2.2% and 6.2% kcals from AS in Year 1 and Year 2, respectively. In Year 1, 36.9% had positive AS exposure, increasing to 98.5% by Year 2. Mean fructose intake was 0.7% and 4.6% of daily energy in Year 1 and Year 2, respectively. There were differences in nutrient intake based on ethnicity, with small to large effect sizes observed across macronutrient and sugars intake comparisons. Compared to non-Hispanic women, Hispanic women consumed more percent energy from carbohydrates (47.8% vs 52.1%, *P* = .01; *d* = −0.47) and protein (14.9% vs 17.0%, *P* < .01; *d* = −0.70), and less from fat (37.5% vs 32.7%, *P* < .01; *d* = 0.67). Hispanic mothers also consumed less AS (13.4% vs 9.9%, *P* = .03; *d* = 0.41). However, the proportion of women exceeding the recommended intake for AS was similar between groups (46.4% vs 45.7%, *P* = .93). Hispanic women consumed less fructose (6.5% vs 4.6%, *P* = .02; *d* = 0.43) during pregnancy compared to non-Hispanic mothers. Hispanic offspring consumed more percent energy from carbohydrates in Year 2 (48.2% vs 50.9%, *P* = .01; *d* = −0.46). Offspring relative intake of AS and fructose in Year 1 and Year 2 was similar between ethnic groups.

Offspring anthropometric characteristics (Table 3) were similar between ethnic groups, except for WC which was higher in Hispanic offspring (46.8 vs 47.9 cm, *P* = .04; *d* = 0.37). Body composition characteristics assessed by DXA are also presented in Table 3. In the full cohort, offspring weighed an average of 12,657 ± 1988 grams, had 30.6 ± 5%fat, 3270 ± 1148 grams total FM, 7214 ± 923 grams FFM, 2065 ± 662 grams peripheral FM, and 1158 ± 428 grams central FM. Compared to non-Hispanic offspring, Hispanic offspring had greater %fat (29% vs 33%, *P* = .02; *d* = −0.74), total FM (2934 g vs 3641 g, *P* = .04; *d* = −1.09), and central FM (1052 g vs 1288 g, *P* = .01; *d* = −0.57).

### 3.3. Intake of AS and fructose and offspring outcomes

Table 4 presents the regression results assessing the relationship between AS and fructose intake and offspring anthropometric outcomes measured at 24 months. Maternal intake did not predict any outcomes. In Year 1, higher AS and fructose intake were associated with a higher WFL *z*-score (AS: *b* = 0.05, *P* = .01; fructose: *b* = 0.21, *P* = .01). Higher fructose intake also predicted higher peripheral SKF (*b* = 0.50, *P* = .02) and approached significance in predicting higher WC (*b* = 0.36, *P* = .07). In Year 2, AS intake approached significance in predicting lower WFL *z*-score (*b* = −0.04, *P* = .08), whereas fructose intake showed no associations. Interactions for ethnicity were detected. The association between Year 1 AS intake and peripheral SKF was stronger in Hispanic offspring (*b* = 0.16, *P* = .10), though not significant. Conversely, the association between Year 1 fructose intake and WFL *z*-score was weaker in Hispanic offspring (*b* = −0.30, *P* = .02). Additionally, Year 1 fructose intake approached significance in predicting a weaker association with peripheral SKF in Hispanic offspring (*b* = −0.64, *P* = .05). No significant associations were found for Year 2 AS intake, but Year 2 fructose intake approached significance in predicting a stronger association with WFL *z*-score in Hispanic offspring (*b* = 0.17, *P* = .06).

Table 5 presents the regression results assessing the relationship between AS and fructose intake and offspring body composition outcomes measured by DXA at 24 months. Maternal intake did not predict any outcomes. In Year 1, higher AS intake predicted higher FM (*b* = 91.65, *P* = .03), FFM (*b* = 109.93, *P* < .01), central FM (*b* = 34.76, *P* = .01), and peripheral FM (*b* = 52.89, *P* = .03). Fructose intake in Year 1 was not associated with DXA outcomes. In Year 2, higher AS intake approached significance in predicting lower %fat (*b* = −0.48, *P* = .05), and higher fructose intake approached significance in predicting greater FM (*b* = 134.52, *P* = .08) and FFM (*b* = 126.45, *P* = .05). Interactions for ethnicity were detected. No associations were found between Year 1 AS intake and DXA outcomes across ethnic groups. However, the associations between Year 2 AS intake and both FFM (*b* = −161.59, *P* = .03) and central FM (*b* = −47.20, *P* = .02) were weaker in Hispanic offspring compared to non-Hispanic offspring. No associations were detected between Year 1 or Year 2 fructose intake and DXA outcomes across ethnic groups.

## 4. Discussion

Our study aimed to understand if ethnicity modified the relationships between AS and fructose intake during the first 1000 days and offspring growth and adiposity at 24 months. Overall, we found that maternal and offspring AS intake deviated from the DGA recommendations. In the full cohort, maternal AS intake exceeded the DGA recommendation of < 10% of daily energy, with Hispanic mothers consuming less AS than non-Hispanic women. Even so, there was no difference in the proportion of non-Hispanic and Hispanic women exceeding the AS recommended intake. This shows that while the average intake was lower for Hispanic women, the issue of excessive AS consumption persisted across both groups. Offspring AS intake in Year 1 and Year 2 was similar between ethnic groups, with nearly all exceeding the recommendation of no AS by Year 2. Despite similar intake levels, the associations between AS and fructose intake and body composition outcomes differed by ethnicity, suggesting potential metabolic differences in how early sugars exposure influences growth. In Hispanic offspring, Year 1 AS intake and Year 2 fructose intake approached significance in predicting stronger associations with peripheral SKF and WFL *z*-scores at 24 months, respectively. Conversely, Year 1 fructose intake was negatively associated with WFL *z*-score and approached significance in predicting a weaker association with peripheral SKF in Hispanic offspring, suggesting that early fructose intake may contribute less to weight gain in this group. DXA measures of body composition revealed additional ethnic differences in response to Year 2 AS intake. Hispanic offspring showed weaker associations between Year 2 AS intake and both FFM and central FM compared to non-Hispanic offspring. This suggests that Hispanic offspring may regulate tissue accrual differently in response to AS exposure in Year 2, particularly by limiting gain in lean mass and central FM. Overall, our findings support that the effects of AS and fructose intake on body composition are dynamic over time and differ by ethnicity. The observed differences align with research suggesting that Hispanic populations may exhibit distinct energy partitioning and growth regulation mechanisms in early childhood, highlighting the importance of considering ethnic differences in metabolic responses to early-life sugars exposure.

Research on how early-life AS and fructose intake influence adiposity is limited in Hispanic populations. Our findings show that these relationships differ by ethnicity and underscore the need for further investigation. In non-Hispanic offspring, AS exposure during pregnancy [8, 9, 11, 12, 30] and toddlerhood [31, 32] is linked to higher BMI and increased adiposity measures. The effects of early fructose exposure on adiposity outcomes in non-Hispanic populations remain largely unexplored in human studies. Furthermore, research on AS and fructose intake during pregnancy and toddlerhood in Hispanic populations is scarce. To our knowledge, only one study assessed the relationship between total sugar intake (vs AS intake) and adiposity outcomes in a Hispanic/Latino population. In overweight Latino youth aged 10 to 17 years old, higher total sugar intake was positively correlated with BMI, BMI *z*-scores, and total FM assessed by DXA [33]. Additionally, fructose intake was positively correlated with total FM [33]. Although our study focused on younger ages, similar patterns emerged. In Hispanic offspring, Year 1 AS intake and Year 2 fructose intake were more strongly associated with peripheral adiposity and weight at 24 months, respectively, compared to non-Hispanic offspring. However, the effects of Year 1 fructose intake on weight and peripheral adiposity and Year 2 fructose intake on FFM and central FM were weaker in Hispanic offspring. These findings suggest that factors beyond diet—such as metabolic programming, lifestyle behaviors, or early-life stressors—may shape these relationships differently across ethnic groups [34]. Additionally, timing of growth trajectories may influence these associations [35]. Rapid growth in infancy could amplify the impact of AS intake on weight gain, while slower growth in the second year may attenuate these effects. The influence of AS and fructose on adiposity also likely depends on overall energy balance, as a positive energy balance is required for adiposity accrual [36]. Contrary to our findings, Goran et al. [37] studied mother-infant pairs who were exclusively breastfeeding and found that higher fructose concentrations in breastmilk were associated with higher fat and lean mass at 6 months old. Given the limited data available, further research is needed to better understand how early dietary exposures to AS and fructose interact with ethnic-specific physiological and environmental factors. Such insights could inform tailored dietary recommendations to mitigate obesity risk across diverse populations.

Our study found that AS and fructose intake during infancy and toddlerhood was associated with weight and body composition measures at 24 months, independent of ethnicity. Specifically, higher AS intake in Year 1 predicted greater WFL *z*-scores, FM, FFM, central FM, and peripheral FM. Additionally, higher fructose intake in Year 1 approached significance in predicting higher WC, while higher fructose intake in Year 2 approached significance in predicting higher FM and FFM. These findings suggest that early sugars exposure may influence body composition trajectories. Although few studies have directly assessed AS intake during infancy and toddlerhood in relation to obesity risk, existing research supports this association. Kong et al. [20] showed that higher AS intake during infancy and toddlerhood was related to rapid weight gain, a well-established risk factor for later obesity. Similarly, studies show that exposure to SSB, a primary source of fructose, before 2 years old is associated with increased obesity risk in childhood [32, 38]. In contrast, we also found that higher AS intake in Year 2 approached significance in predicting lower WFL and %fat at 24 months. This differs from prior findings showing that increased AS intake during toddlerhood was associated with a higher BMI at 7 years old [31]. One possible explanation for this discrepancy is the influence of early growth trajectories. Rapid weight gain in infancy is often followed by a period of slowed growth in the second year of life [35]. If early AS exposure contributes to faster weight gain in Year 1, metabolic adaptations in Year 2 may regulate growth and fat accrual differences, potentially explaining why AS intake in Year 2 did not correspond with increased adiposity. Overall, our findings contribute to the growing body of evidence supporting that early-life AS exposure may have lasting implications for weight status, but the effects may depend on timing and individual growth patterns. Further research is needed to explore how early AS intake interacts with metabolic programming and growth regulation across different developmental stages.

As stated above, our study found that higher AS and fructose exposure during infancy and toddlerhood predicted an overall heavier weight status at 24 months, that is, increases in both FM and FFM measures. These positive relationships suggest that AS are contributing to increases in fat accrual but also lean mass accrual. Increases in BMI in early childhood are mainly explained by increases in FFM, rather than FM [39–41]. Therefore, the more robust body composition measures used in our study may have captured increases in other body compartments that have not been previously assessed in relation to AS and fructose exposure. Lean mass and peripheral FM are protective to metabolic health [42–44]. However, it is unknown if they retain their protective properties in the presence of excess total and central adiposity. It will be important for future studies to confirm our findings and assess how a concurrent increase in FM and FFM measures in early childhood impacts obesity risk and metabolic health.

In addition to a lack of data, another barrier to comparing existing studies is the type of sugar variable assessed; some studies report AS while other studies report total sugars. Total sugars represent AS plus any sugar naturally present in foods (e.g., intrinsic sugars in fruits, vegetables, and dairy). While total sugars and AS capture different types of sugars found in foods, they are highly correlated. In toddlerhood and childhood, a high proportion of total sugars comes from foods high in AS (e.g., SSB, cookies, cakes) vs fruits or dairy products high in intrinsic sugars. The majority of toddlers (> 98%) consume AS on a given day [7, 20]. Data are lacking on the percentage of youth who consume AS daily, but 66% of boys and girls consume SSB daily comprising over 7% of their total daily caloric intake [45]. It is likely that intrinsic sugars and AS relate differently to health outcomes. For example, food groups like fruit contain other nutrients (e.g., fiber, vitamins, minerals) that are known to be positively related to health outcomes; whereas a diet high in AS is related to lower dietary quality resulting in a displacement of nutritious foods [46].

Ethnic differences in AS intake are important for interpreting our findings. While previous research reports that Hispanics often exceed AS intake recommendations [47–49], intake is generally lower than that of non-Hispanic Black and White populations [7, 21, 22, 50]. Similarly, we found that Hispanic mothers consumed less AS and fructose compared to non-Hispanic mothers. However, despite these differences, a comparable proportion of Hispanic and non-Hispanic women exceeded recommended AS intake levels. AS intake was also slightly lower among Hispanic offspring, though not significantly. Excessive AS intake remained a concern across all groups. Differences in how sugars are introduced during the first 2 years of life may contribute to ethnic disparities in metabolic outcomes. Ethnicity-specific metabolic patterns may be influenced by genetic predispositions that affect sugar metabolism and storage [51]. For example, Davis et al. [52], found that higher sugar intake in Hispanic children was associated with changes in hepatic fat deposition, suggesting differences in fat metabolism and storage. Additionally, epigenetic modifications driven by prenatal and early-life exposures may contribute, as supported by studies linking maternal diet to offspring metabolic programming [53, 54]. Neurodevelopmental factors, such as variations in reward pathway activation in response to sugar intake, may also influence appetite regulation and body composition differently in Hispanic children compared to non-Hispanic children [55, 56]. The early emergence of distinct metabolic profiles in Hispanic offspring could have long-term implications for obesity risk and metabolic health. Our results suggest that AS and fructose intake during infancy and toddlerhood may shape metabolic responses differently in Hispanic children, potentially predisposing them to a unique metabolic phenotype [44, 57]. This differential response to early sugars exposure underscores the need for further research to determine if these relationships persist and evolve over time.

Our study has many strengths, including a prospective longitudinal design across two NIH-funded trials. The cohort included both Hispanic and non-Hispanic mother-child dyads who were followed from pregnancy to when the offspring were 24 months of age. Research to-date assessing AS intake in the maternal and child population has mainly used weight and/or BMI as primary outcomes, both of which are not the most accurate in measuring body composition in growing children [58]. Therefore, a strength of this study was the use of more robust measures of adiposity including circumference and SKF measures, and the DXA, which is a validated tool providing precise and accurate measures of total FM and FM distribution [59, 60]. Another strength was the use of validated and accurate tools to assess prenatal and offspring dietary intake [61–65].

Our study also has limitations. First, only one offspring dietary recall was collected at each time point, whereas at least two recalls are typically recommended to accurately reflect usual intake over a given period [29]. We performed a principal component analysis to understand if AS intake cohered between the 2-week and 6-month and the 12- and 24-month time periods. We found no difference between each time period. Therefore, we averaged the 2-week and 6-month recalls to represent infant nutrition (predominantly breastmilk and/or formula) and the 12- and 24-month recalls to represent toddler nutrition (when solid foods are the primary source of intake). A limitation in assessing prenatal dietary intake was the potential influence of nausea and vomiting (e.g., morning sickness). Symptoms typically begin between 4 and 6 weeks of gestation, peak between 8 and 12 weeks, and resolve by 20 weeks [66]. In our sample, prenatal dietary intake was assessed on average at 16.4 weeks, minimizing the impact of early pregnancy symptoms on reported intake. However, residual effects of early symptoms or individual variation in symptom resolution may have influenced dietary patterns in some participants. A potential limitation of this study’s prenatal dietary assessment is the use of two different methods to collect dietary intake. The ECHO trial reported differences in dietary intake estimates derived from recall and FFQ data [67]. In the ADORE study, both the DHQ-II and dietary recall data identified a similar proportion of women with dietary intake below the Dietary Reference Intakes, excluding niacin, thiamin, and vitamin B6 [68]. However, it is unknown if the differences arise from the assessment method or variations in the nutrient content of foods consumed by the participants. Given this limitation, for all models assessing the relationship between maternal AS and fructose intake and offspring outcomes, only the main effect of maternal intake was examined and not the interaction with ethnicity. Even so, this limitation should be considered when interpreting our results. A notable gap in the literature is the lack of validated FFQs designed for diverse populations, allowing consistent use of a single intake method. Studies should focus on developing such tools to address these important research questions in diverse samples of pregnant women.

Diet quality, including SSB intake, can differ by ethnic subgroups [69]. However, our study was unable to distinguish between Hispanic and Latinx subgroups, as participants self-identified only as Hispanic or non-Hispanic. Additionally, there were descriptive differences between Hispanic and non-Hispanic participants, including education level, income, and GWG. Household education level and income can be sociodemographic predictors of higher AS intake [70]. Amount [71] and timing [72] of GWG can influence offspring obesity risk. These variables were considered as covariates in our models and retained if they statistically contributed to predicting outcomes.

Lastly, this article reports an exploratory analysis secondary to the primary goal of the study, therefore, the study was not powered to answer the aims of this article. The DXA results were comprised from a subgroup analysis of toddlers who successfully completed a DXA scan and dietary intake data were available (*n* = 42). Given this is a subgroup and secondary analysis with a limited sample size, results should be interpreted with caution. Future studies are needed to confirm our findings.

## 5. Conclusion

Poor nutrition in the first 1000 days impacts child health outcomes. Studies support a positive association between early AS exposure and obesity risk. Intake of AS, including fructose, is lower or comparable among Hispanic children compared to non-Hispanic children. Yet, Hispanic children experience higher rates of obesity, which suggests a potential sensitivity in Hispanics to AS exposure. Studies have not assessed how ethnicity modifies the relationship between AS and fructose intake during pregnancy, infancy, and toddlerhood and offspring adiposity. We found that the timing of AS and fructose exposure during infancy and toddlerhood predicted changes in body composition at 24 months, and outcomes varied by ethnicity. Further research is warranted to confirm our findings and understand if relationships persist and impact later obesity risk and metabolic health.

## Figures and Tables

**Table 1 –:** Descriptive characteristics of a healthy cohort of non-Hispanic and Hispanic mother-child pairs.

*Maternal descriptive characteristics*				
	Total (*n* = 130)	Non-Hispanic (*n* = 84)	Hispanic (*n* = 46)	*P*-value
Age (y)	31.39 ± 5.42	31.88 ± 5.05	30.48 ± 5.98	.16
Education, *n* (%)				**<.01**
High school/GED	**42 (32.3)**	**12 (14.3)**	**30 (65.2)**	
Some college	**21 (16.2)**	**16 (19.0)**	**5 (10.9)**	
Bachelor’s degree	**37 (28.5)**	**28 (33.3)**	**9 (19.6)**	
Graduate school	**30 (23.1)**	**28 (33.3)**	**2 (4.3)**	
Income, *n* (%)				**<.01**
<$50,000	**62 (47.7)**	**24 (28.6)**	**38 (82.6)**	
$50K to < $100K	**25 (19.2)**	**19 (22.6)**	**6 (13.0)**	
>$100K to <$150K	**26 (20)**	**26 (31)**	**0 (0)**	
>$150K	**17 (13.1)**	**15 (17.9)**	**2 (4.3)**	
Race, *n* (%)				*.07*
White	*97 (74.6)*	*67 (79.8)*	*30 (65.2)*	
Other than White	*33 (25.4)*	*17 (20.2)*	*16 (34.8)*	
Ever smoker, *n* (%)	31 (23.8)	22 (26.2)	9 (19.6)	.40
Marital status, *n* (%)				.55
Married/partnered	94 (72.3)	62 (73.8)	32 (69.6)	
Separated/divorced	5 (3.8)	4 (4.8)	1 (2.2)	
Unmarried/single	31 (23.8)	18 (21.4)	13 (28.3)	
DHA Tx group, *n* (%)				.86
200 mg/d	58 (44.6)	37 (44.0)	21 (45.7)	
1000 mg/d	72 (55.4)	47 (56.0)	25 (54.3)	
Parity, *n* (%)				**.01**
Nulliparous	**51 (39.2)**	**41 (48.8)**	**10 (21.7)**	
Parous	**79 (60.8)**	**43 (51.2)**	**36 (78.3)**	
Prepregnancy BMI	28.23 ± 7.07	28.55 ± 7.28	27.64 ± 6.72	.49
Prepregnancy BMI, *n* (%)				.81
Healthy	49 (37.7)	31 (36.9)	18 (39.1)	
Overweight	38 (29.2)	27 (32.1)	11 (23.9)	
Obese	43 (33.1)	26 (31.0)	17 (37.0)	
GWG (kg)	**13.73 ± 7.57**	**15.65 ± 6.61**	**10.22 ± 8.01**	**<. 01**
GWG, *n* (%)				**<. 01**
Not excessive	**59 (45.4)**	**28 (33.3)**	**31 (67.4)**	
Excessive	**71 (54.6)**	**56 (66.7)**	**15 (32.6)**	
GDM or HDP Dx, *n* (%)	19 (14.6)	14 (16.7)	5 (10.9)	.38
*Offspring descriptive characteristics*				
	Total (*n* = 130)	Non-Hispanic (*n* = 80)	Hispanic (*n* = 50)	*P*-value
Age at visit (mo)	24.59 ± 0.69	24.55 ± 0.66	24.66 ± 0.73	.40
Sex, *n* (%)				1.00
Male	65 (50.0)	40 (50.0)	25 (50.0)	
Female	65 (50.0)	40 (50.0)	25 (50.0)	
Race, *n* (%)				**.04**
White	**87 (66.9)**	**59 (73.7)**	**28 (56.0)**	
Other than White	**43 (33.1)**	**21 (26.3)**	**22 (44.0)**	
GA at birth (wk)	38.74 ± 1.59	38.76 ± 1.74	38.71 ± 1.33	.85
Gestation length, *n* (%)				.92
Full-term	120 (92.3)	74 (92.5)	46 (92.0)	
Preterm (<37 wk)	10 (7.7)	6 (7.5)	4 (8.0)	
Birth weight (grams)	3260.7 ± 497.5	3272.5 ± 529.0	3241.7 ± 447.2	.73
Birth weight percentile (%)	49.4 ± 26.7	50.8 ± 27.2	47.2 ± 26.0	.46
Birth length (cm)	50.0 ± 2.6	50.1 ± 2.6	49.8 ± 2.4	.49
Birth WFL percentile	40.1 ± 30.0	38.3 ± 29.5	42.8 ± 31.0	.42
Birth WFL z-score	−0.4 ± 1.2	−0.4 ± 1.1	−0.3 ± 1.3	.60

Abbreviations: DHA, docosahexaenoic acid; Dx, diagnosis; GA, gestational age; GDM, gestational diabetes mellitus; GWG, gestational weight gain; HDP, hypertensive disorder of pregnancy; Tx, treatment; WFL, weight-for-length.

***P* < .05**, *.05* ≤ *P* ≤ *.10*. Values expressed as mean ± SD unless otherwise noted.

**Table 2 –:** Dietary characteristics of a healthy cohort of non-Hispanic and Hispanic mother-child pairs.

*Maternal dietary characteristics*						
		Total (*n* = 130)	Non-Hispanic (*n* = 84)	Hispanic (*n* = 46)	*P*-value	Effect size
Total energy (kcals)		1904 ± 666	1897 ± 709	1916 ± 586	.88	−0.03
Carbohydrates (% kcal)		**49.3 ± 9.2**	**47.8 ± 9.7**	**52.1 ± 7.7**	**.01**	**−0.47**
Fat (% kcal)		**35.8 ± 7.5**	**37.5 ± 7.3**	**32.7 ± 6.8**	**<. 01**	**0.67**
Protein (% kcal)		**15.6 ± 3.2**	**14.9 ± 3.1**	**17.0 ± 3.0**	**<. 01**	**−0.70**
Added sugars (grams)		*61.1* ± 59.1	*68.2* ± *68.9*	*48.1* ± *31.7*	*.06*	*0.34*
Added sugars (% kcal)		**12.1 ± 8.7**	**13.4 ± 9.9**	**9.9 ± 5.3**	**.03**	**0.41**
Below/above guidelines, *n* (%)					.93	0.01
<10% kcal from AS		70 (53.8)	45 (53.6)	25 (54.3)		
≥10% kcal from AS		60 (46.2)	39 (46.4)	21 (45.7)		
Fructose (grams)		**28.5 ± 29.6**	**32.6 ± 35.4**	**21.0 ± 11.0**	**.03**	**0.40**
Fructose (% kcal)		**5.8 ± 4.5**	**6.5 ± 5.3**	**4.6 ± 2.1**	**.02**	**0.43**
Fruit Servings (cup eq)		0.7 ± 0.6	0.7 ± 0.6	0.7 ± 0.4	.79	0.05
*Offspring dietary characteristics*						
		Total (*n* = 130)	Non-Hispanic (*n* = 80)	Hispanic (*n* = 50)	*P*-value	Effect size
BF duration (mo)		3.9 ± 3.3	3.7 ± 3.2	4.1 ± 3.5	.63	−0.16
Feeding mode at 6 mo					.32	0.24
All/mostly BF		68 (52.3)	47 (58.8)	21 (42.0)		
Equal BF and formula		20 (50.4)	7 (8.8)	13 (26.0)		
All/mostly formula		42 (32.3)	26 (32.5)	16 (32.0)		
Feeding mode at 12 mo					.69	0.04
All/mostly BF		50 (38.5)	32 (40)	18 (36.0)		
Equal BF and formula		10 (7.7)	6 (7.5)	4 (8.0)		
All/mostly formula		70 (53.8)	42 (52.5)	28 (56.0)		
Age of solid food introduction (mo)		5.2 ± 1.4	5.2 ± 1.4	5.2 ± 1.2	.97	−0.01
Total energy (kcals)	Y 1	604 ± 115	602 ± 113	606 ± 120	.86	−0.03
	Y 2	1022 ± 273	1050 ± 257	976 ± 294	.14	0.27
Carbohydrates (% kcal)	Y 1	*41.6* ± *3.5*	*41.1* ± *3.1*	*42.4* ± *4.0*	*.05*	*−0.36*
	Y 2	**49.3 ± 6.0**	**48.2 ± 6.4**	**50.9 ± 5.1**	**.01**	**−0.46**
Fat (% kcal)	Y 1	*50.7* ± *3.9*	*51.2* ± *3.7*	*49.9* ± *4.2*	*.06*	*0.34*
	Y 2	*36.8* ± *5.6*	*37.5* ± 5.7	*35.5* ± 5.2	*.05*	*0.36*
Protein (% kcal)	Y 1	7.4 ± 1.1	7.3 ± 1.2	7.5 ± 1.0	.47	−0.13
	Y 2	13.8 ± 3.2	14.1 ± 3.1	13.4 ± 3.4	.12	0.23
Added sugars (grams)	Y 1	3.8 ± 8.5	4.1 ± 8.7	3.3 ± 8.2	.60	0.10
	Y 2	17.2 ± 14.0	18.7 ± 13.2	14.8 ± 15.0	.13	0.28
Added sugars (% kcal)	Y 1	2.2 ± 4.9	2.3 ± 4.9	2.1 ± 4.8	.83	0.04
	Y 2	6.2 ± 4.2	6.7 ± 4.0	5.5 ± 4.5	.12	0.28
AS exposure, *n* (%)	Y 1	48 (36.9)	28 (35.0)	20 (40.0)	.57	0.05
	Y 2	*128 (98.5)*	*80 (100)*	*48 (96.0)*	*.07*	*0.16*
Fructose (grams)	Y 1	*1.1* ± *2.3*	*0.8* ± *1.8*	*1.5* ± *2.8*	*.10*	*−0.30*
	Y 2	*11.9* ± *7.2*	*12.8* ± *7.1*	*10.5* ± *7.2*	*.08*	*0.32*
Fructose (% kcal)	Y 1	0.7 ± 1.5	0.5 ± 1.4	0.9 ± 1.5	.18	−0.24
	Y 2	4.6 ± 2.3	4.8 ± 2.5	4.2 ± 2.0	.14	0.27
Fruit Servings (cup eq)	Y 1	*0.1* ± *0.2*	*0.1* ± *0.2*	*0.2* ± *0.3*	*.06*	*−0.35*
	Y 2	0.8 ± 0.6	0.8 ± 0.6	0.8 ± 0.5	.53	0.11

Abbreviations: AS, added sugars; BF, breastfeeding; Y1, Year 1; Y2, Year 2.

***P* < .05**, *.05* ≤ *P* ≤ *.10*. Values expressed as mean ± SD unless otherwise noted. Fruit servings are presented as cup equivalents. Mothers’ dietary intake was collected between 12 and 20 weeks gestation. Offspring dietary intake was collected at 2 weeks, 6 months, 12 months, and 24 months. Offspring dietary intake was averaged between 2 weeks and 6 months to represent the infancy period (Year 1), and between 12 months and 24 months to represent the toddlerhood period (Year 2).

**Table 3 –:** Anthropometric and body composition characteristics of a healthy cohort of non-Hispanic and Hispanic offspring at 24 months.

*Anthropometric characteristics*					
	Total (*n* = 130)	Non-Hispanic (*n* = 80)	Hispanic (*n* = 50)	*P*-value	Effect size
Weight (kg)	*12.6* ± *1.6*	*12.4* ± *1.2*	*12.9* ± *2.0*	*.07*	*−0.34*
Length (cm)	86.1 ± 3.0	86.0 ± 2.7	86.3 ± 3.5	.53	−0.11
Waist circumference (cm)	**47.2 ± 3.2**	**46.8 ± 2.7**	**47.9 ± 3.8**	**.04**	**0.37**
Weight *z*-score	0.2 ± 1.0	0.1 ± 0.9	0.4 ± 1.2	.15	−0.26
Length *z*-score	−0.1 ± 0.9	−0.1 ± 0.8	−0.02 ± 1.0	.56	−0.11
WFL *z*-score	0.5 ± 1.1	0.4 ± 1.0	0.7 ± 1.2	.12	−0.28
Central SKF (mm)	10.6 ± 2.8	10.6 ± 2.7	10.5 ± 3.0	.85	0.03
Peripheral SKF (mm)	11.7 ± 2.6	11.7 ± 2.5	11.7 ± 2.9	.96	0.01
Total SKF (mm)	66.5 ± 15.7	66.5 ± 14.7	66.5 ± 17.3	.98	0.01
*DXA characteristics*					
	Total (*n* = 42)	Non-Hispanic (*n* = 22)	Hispanic (*n* = 20)	*P*-value	Effect size
Weight (g)	12,657.1 ± 1987.7	12,295.5 ± 1089.8	13,055.0 ± 2625.8	.22	−0.39
%fat (%)	**30.6 ± 5.3**	**28.8 ± 5.1**	**32.5 ± 4.9**	**.02**	**−0.74**
Total FM (g)	**3270.4 ± 1147.5**	**2933.5 ± 664.8**	**3641.0 ± 1441.1**	**.04**	**−1.09**
Total FFM (g)	7214.3 ± 923.0	7167.6 ± 640.7	7265.7 ± 1174.4	.74	−0.44
Peripheral FM (g)	2065.4 ± 662.2	1907.8 ± 405.3	2230.7 ± 833.1	.12	−0.50
	**Total (*n* = 85)**	**Non-Hispanic (*n* = 47)**	**Hispanic (*n* = 38)**	***P*-value**	**Effect size**
Central FM (g)	**1157.8 ± 428.3**	**1052.3 ± 257.3**	**1288.2 ± 550.0**	**.01**	**−0.57**

Abbreviations: %fat, percentage body fat; biceps, thigh, triceps, peripheral SKF; DXA, dual-energy x-ray absorptiometry; FFM, fat-free mass; FM, fat mass; SKF, skinfold; suprailiac, subscapular, flank, central SKF; WFL, weight-for-length.

***P* < .05**, *.05* ≤ *P* ≤ *.10*. All values presented as mean ± SD.

**Table 4 –:** Intake of AS and fructose in the first 1000 days and offspring anthropometric outcomes at 24 months old (*n* = 130).

Independent variables	Dependent Variables							
	WFL		WC^a^		Central SKF^b^		Peripheral SKF^b,c^	
	*β*	*P*	*β*	*P*	*β*	*P*	*β*	*P*
*Added sugars*								
Ethnicity	0.28	.15	0.99	*.09*	0.40	.51	0.85	.15
Mat intake	0.01	.65	−0.03	.33	0.01	.69	0.02	.39
Y1 intake	0.05	.01	0.08	.18	0.08	.13	0.07	.24
Y2 intake	−0.04	*.08*	−0.09	.19	−0.02	.75	−0.09	.14
Y1*Ethnicity	-	-	-	-	-	-	0.16	*.10*
Y2*Ethnicity	-	-	-	-	-	-	-	-
*Fructose*								
	WFL^a^		WC^a,d^		Central SKF^b^		Peripheral SKF^b^	
	*β*	*P*	*β*	*P*	*β*	*P*	*β*	*P*
Ethnicity	0.34	.08	1.30	.03	0.19	.73	0.30	.57
Mat Intake	0.01	.90	−0.01	.85	0.02	.73	0.01	.98
Y1 Intake	0.21	.01	0.36	*.07*	0.26	.13	0.50	.02
Y2 Intake	−0.03	.54	0.12	.34	−0.11	.29	−0.03	.76
Y1*Ethnicity	−0.30	.02	-	-	-	-	−0.64	*.05*
Y2*Ethnicity	0.17	*.06*	-	-	-	-	-	-

Abbreviations: Maternal, Mat; SKF, skinfold; WC, waist circumference; WFL, weight-for-length *z*-score; Y1, Year 1; Y2, Year 2.

***P* < .05**, *.05* ≤ *P* ≤ *.10*. A dash (−) represents removal of a nonsignificant interaction term. Covariates retained in each model following backward selection (*P* < .10) are listed below.

a Prepregnancy BMI.

b Total gestational weight gain.

c Maternal education.

d Offspring race (White or non-White).

**Table 5 –:** Intake of AS and fructose in the first 1000 days and offspring DXA outcomes at 24 months old (*n* = 42).

Independent variables	Dependent variables									
	%fat^a^		FM		FFM^b,c^		Central FM (*n* = 85)		Peripheral FM	
	*β*	*P*	*β*	*P*	*β*	*P*	*β*	*P*	*β*	*P*
*Added sugars*										
Ethnicity	4.77	.02	457.39	.21	−161.36	.55	221.64	.01	166.85	.43
Mat intake	0.14	.23	−5.53	.80	−8.62	.64	−7.06	.26	−2.95	.82
Y1 intake	0.03	.90	91.65	.03	109.93	<. 01	34.76	.01	52.89	.03
Y2 intake	−0.48	*.05*	−59.30	.22	42.59	.30	6.48	.66	−40.54	.15
Y1*Ethnicity	-	-	-	-	-	-	-	-	-	-
Y2*Ethnicity	-	-	-	-	−161.59	.03	−47.20	.02	-	-
*Fructose*										
	%fat		FM^d^		FFM^d^		Central FM (*n* = 85)		Peripheral FM^d^	
	β	*P*	β	*P*	β	*P*	β	*P*	β	*P*
Ethnicity	4.08	.02	544.35	.11	−176.77	.53	251.25	.01	232.41	.26
Mat intake	0.02	.92	−9.74	.81	−34.68	.29	8.58	.51	−9.94	.68
Y1 intake	−0.64	.31	−128.16	.32	9.17	.93	4.87	.89	−80.34	.30
Y2 intake	0.34	.39	134.52	*.08*	126.45	*.05*	21.85	.32	70.96	.13
Y1*Ethnicity	-	-	-	-	-	-	-	-	-	-
Y2*Ethnicity	-	-	-	-	-	-	-	-	-	-

Abbreviations: %fat, percentage body fat; FFM, fat-free mass; FM, fat mass; Maternal, Mat; Y1, Year 1; Y2, Year 2.

***P* < .05**, *.05* ≤ *P* ≤ *.10*. A dash (−) represents removal of a nonsignificant interaction term. Covariates retained in each model following backward selection (*P* < .05) are listed below.

a Total gestational weight gain.

b Prepregnancy BMI.

c Ever smoker (yes or no).

d Child age at time of measurement (months).

## Abbreviations:

%fat: percentage body fat
AS: added sugars
BMI: body mass index
DGA: Dietary Guidelines for Americans
DHA: docosahexaenoic acid
DHQ: Diet History Questionnaire
DXA: dual-energy X-ray absorptiometry
FFM: fat-free mass
FM: fat mass
GDM: gestational diabetes mellitus
GWG: gestational weight gain
HDP: hypertensive disorder of pregnancy
NDSR: Nutrition Data System for Research
SKF: skinfold
SSB: sugar-sweetened beverages
WC: waist circumference
WFL: weight-for-length
